# Recognizing multiple epiphyseal dysplasia in children presenting with joint pain: a commonly overlooked skeletal dysplasia

**DOI:** 10.1007/s00431-025-06176-8

**Published:** 2025-05-20

**Authors:** Tuğba Daşar, Gözde İmren, Adalet Elçin Yıldız, Gizem Ürel Demir, Gülen Eda Utine, Güney Yılmaz, Pelin Özlem Şimşek Kiper

**Affiliations:** 1 Genetics Division, Department of Pediatric Basic Sciences, Institute of Child Health, Hacettepe University, Ankara, Turkey; 2 Division of Pediatric Genetics, Department of Pediatrics, Ankara Sincan Training and Research Hospital, Ankara, Turkey; 3 Department of Medical Genetics, Faculty of Medicine, Hacettepe University, Ankara, Turkey; 4 Department of Radiology, Faculty of Medicine, Hacettepe University, Ankara, Turkey; 5 Division of Pediatric Genetics, Department of Pediatrics, Faculty of Medicine, Hacettepe University, Ankara, Turkey; 6 Department of Orthopedics, Faculty of Medicine, Hacettepe University, Ankara, Turkey

**Keywords:** MED, Multiple epiphyseal dysplasia, Skeletal dysplasia, Joint pain

## Abstract

Multiple epiphyseal dysplasias are relatively common skeletal disorders, and diagnosing children can often be challenging due to various presenting complaints, including joint pain, short stature, waddling gait, joint deformities, and myopathy findings. Patients may experience early-onset osteoarthritis, and in some cases, joint replacement therapy may be required. Radiographs are characterized by flat, small, and irregularly shaped epiphyses, especially in the hips and knees. Multiple epiphyseal dysplasias are caused by variants in the genes encoding important cartilage extracellular matrix proteins, enzymes, and transporter proteins, including *COMP*, *MATN3*, *COL9A1*, *COL9A2*, *COL9A3*, *CANT1*, and *SLC26A2*. We aimed to investigate the clinical, radiographic, and molecular findings, along with the natural course of the disease, in a group of patients with multiple epiphyseal dysplasia. The children with a clinical diagnosis of multiple epiphyseal dysplasia and their affected parents registered at our center over a period of 20 years were evaluated. The clinical and radiographic findings were reviewed. The genetic test was performed whenever possible, with the aid of Sanger sequencing or exome sequencing as appropriate. A total of 27 patients (21 children and six affected parents) from 14 unrelated families were clinically diagnosed with multiple epiphyseal dysplasia. The genetic etiology could be revealed in 25 patients (*n* = 25/27, 92.5%) from 12 unrelated families. Of the 25 patients, 16 (64%) were male and nine (36%) were female. The age at genetic diagnosis ranged from 4 to 50 years, with a median age of 10 years. Nine patients (9/25, 36%) had short stature, 17 (17/25, 68%) experienced joint pain, and seven (7/25, 28%) required orthopedic surgery. The most frequent complaints leading to referral were joint pain and difficulty walking. Genetic tests revealed a total of 12 variants in 12 families, among which three were novel: *COMP* (13/25 patients, 52%; 7/12 families, 58.3%), *MATN3* (5/25 patients, 20%; 2/12 families, 16.6%), *SLC26A2* (5/25 patients, 20%; 2/12 families, 16.6%), and *COL9A2* (2/25 patients, 8%; 1/12 families 8.3%). Of the patients who underwent orthopedic surgery (*n* = 7), five had *COMP* variants. Patients with *COMP* variants exhibited a more severe phenotype, consistent with the literature.

*Conclusion*: Multiple epiphyseal dysplasias represent a genetically heterogeneous group of disorders that may present clinical and diagnostic challenges. This condition should be considered when evaluating patients who experience joint pain and have radiographic findings suggestive of Perthes disease. A comprehensive skeletal survey and genetic tests are essential for the accurate diagnosis and management of this condition.

**Table:** 

**What is Known**: • *MED represents one of the most prevalent categories of skeletal dysplasias, presenting clinically with progressive joint pain, skeletal deformities, gait disturbances, or features suggestive of an underlying myopathy.*	
**What is New**: • *In this study, we present 25 patients with MED, identify three novel variants, establish significant correlations between genotype and phenotype, and demonstrate how genetic analysis facilitates the differentiation of MED subtypes, thereby offering clearer insights into the genetic foundations and clinical implications of this condition.*

**What is Known**:

• *MED represents one of the most prevalent categories of skeletal dysplasias, presenting clinically with progressive joint pain, skeletal deformities, gait disturbances, or features suggestive of an underlying myopathy.*

**What is New**:

• *In this study, we present 25 patients with MED, identify three novel variants, establish significant correlations between genotype and phenotype, and demonstrate how genetic analysis facilitates the differentiation of MED subtypes, thereby offering clearer insights into the genetic foundations and clinical implications of this condition.*

## Introduction

The genetic skeletal disorders are individually rare, but collectively rather common, with an approximate incidence of 1 in 5000 [1]. Among them, multiple epiphyseal dysplasias (MEDs) represent a genetically heterogeneous group primarily affecting the epiphysis of long bones, particularly in the hips and knees [2–4]. According to the Nosology of Genetic Skeletal Disorders 2023 revision, MED is classified within Group 9, “Pseudoachondroplasia and the multiple epiphyseal dysplasias” [5]. To date, seven genes have been demonstrated in the etiology of MED, involving both recessive and dominant inheritance. Monoallelic variants in *COMP*, *COL9A2*, *COL9A3*, *COL9A1*, and *MATN3* have been linked to types 1 (OMIM# 132400), 2 (OMIM# 600204), 3 (OMIM# 600969), 6 (OMIM# 614135), and 5 (OMIM# 607078) MED, respectively. On the other hand, biallelic variants in *SLC26A2* are responsible for type 4 MED (OMIM# 226900) [2–5]. Notably, a recent addition to the classification is a new type of MED (Type 7, OMIM# 617719), identified in two families and associated with biallelic variants in the CANT1 gene [6], which is also implicated in Desbuquois dysplasia. However, it is important to emphasize that patients with MED Type 7 do not display the typical clinical features associated with Desbuquois dysplasia, including characteristic facial dysmorphism, joint dislocations, short extremities, coronal clefts, or accessory ossification centers in the hands, findings commonly observed in individuals with this condition.

The clinical diagnosis of MED may be challenging since the presenting signs are often rheumatological or neurological, such as joint pain, walking difficulty, and myopathy. The disproportionate short stature, which, in general, is a major finding in skeletal dysplasias, is usually not present. Nevertheless, patients with MED may display short stature. Patients usually present with joint pain following physical activity, often manifesting in early childhood along with early-onset osteoarthritis, which frequently necessitates joint replacement. In addition, a waddling gait, limited joint mobility, and joint deformities are also seen [3, 7, 8]. Radiographic examination commonly reveals delayed epiphyseal ossification, as well as small, flat, and deformed epiphyses, particularly in the hips and knees [9]. The most common deformities include genu valgum/varum in the lower extremities, acetabular dysplasia, coxa vara, and epiphyseal irregularities in the femoral head. These deformities may benefit from orthopedic procedures, including pelvic osteotomies, corrective long bone osteotomies, and guided growth (hemiepiphysiodesis) techniques [10–13]. In this study, we aimed to review the clinical, radiographic, and molecular findings in a group of patients with MED from a single tertiary centre in Turkiye in order to increase the awareness of this entity among clinicians and orthopedic surgeons.

## Methods

The study protocol was approved by the Hacettepe University Ethics Committee (GO 17/321–19) and followed the principles of the Declaration of Helsinki. Informed consent was obtained from all participants and their legal guardians. The study was supported by the Hacettepe University Scientific Research Projects Coordination Unit with Project ID TSA-2017–14392.

### Patients and samples

Patients with a clinical diagnosis of MED who were under follow-up at Hacettepe University Pediatric Genetics Department were included in the study. The patients were enrolled over a span of 20 years, from January 2004 to January 2025. We collected comprehensive clinical data from 25 patients, including demographics (age, sex), medical history, and presenting symptoms such as joint pain, mobility issues, height discrepancies, difficulty walking, challenges in climbing stairs, muscle weakness, and leg deformities. Physical examination findings were documented, including joint range of motion and short stature, along with any surgical history related to joint or skeletal interventions. Radiographic data were obtained through standard anteroposterior and lateral X-ray views of affected joints (hands, feet, hips, knees, ankles), assessing characteristic findings such as delayed ossification of epiphyses, irregular contours of carpal and tarsal bones, flat or rounded epiphyses, irregularities in the epiphyses, the presence of a “glacier crevice” sign or “harlequin hat” appearance at the knees, changes in bone age compared to chronological age, and evaluation for coxa deformities and genu deformities.

### Molecular analysis

Genomic DNA was extracted from the peripheral blood sample of the patients and their parents using the QIAamp DNA Blood Mini Kit (Qiagen Valencia, CA). Firstly, Sanger sequencing was performed in some patients in order to analyze coding regions and intron boundaries of the COMP gene, using the ABI 3500 Genetic Analyzer (Applied Biosystems, Foster City, CA, USA). Exome sequencing was performed in patients with no variant in the COMP gene and in patients subsequently included in the study cohort.The Ion Ampliseq Exome kit was used for library preparation. Then the libraries were sequenced in the Ion Proton (Thermo Scientific) and Illumina Next-Seq 550 instruments according to the manufacturer’s instructions. The.vcf files were generated and used for variant interpretation. For filtering, ExAC, dbSNP, and in-house data were used, and common variants with a minor allele frequency > 0.01 were filtered out and the skeletal dysplasia panel (*n* = 552 genes) was applied for further filtering.

Pathogenicity was assessed using the American College of Medical Genetics and Genomics (ACMG)/Association for Molecular Pathology (AMP) guidelines [14] for the interpretation of sequence variants, which includes population data, computational and predictive data using various lines of computational evidence (CADD, Polyphen) and segregation data [14, 15]. Publicly available software and databases, including the Combined Annotation Dependent Depletion (CADD) database (https://cadd.gs.washington.edu/) [16], Polymorphism Phenotyping v2 (PolyPhen-2, http://genetics.bwh.harvard.edu/pph2) [17], and Mutation Taster (http://www.mutationtaster.org/) [18], were used to assess the clinical significance of the identified novel variants. The three novel variants and one unsubmitted *COL9A2* variant were submitted to ClinVar (*COMP*, c.1303G > C: SCV004814169; *COMP* c.1854G > C: SCV004814168; *MATN3* c.280G > A: SCV004814170; *COL9A2* c.186 + 6 T > G: SCV006052425).

The secondary structure of the COMP, MATN3, and SLC26A2 was obtained from the UniProt database (https://www.uniprot.org/) for schematic representation. Multiple-sequence alignment of COMP and MATN3 was obtained in different species, including *Homo sapiens*, *Pan troglodytes*, *Macaca mulatta*, *Canis lupus familiaris*, *Mus musculus*, *Rattus norvegicus*, *Xenopus tropicalis*, and *Danio rerio*, using the Clustal Omega (https://www.ebi.ac.uk/jdispatcher/msa/clustalo) [19].

## Results

A total of 27 patients from 14 unrelated families were clinically diagnosed with multiple epiphyseal dysplasia. Among them, genetic etiology was identified in 25 patients (*n* = 25/27, 92.5%) from 12 unrelated families using either Sanger sequencing or exome sequencing.

**Fig. 1:**
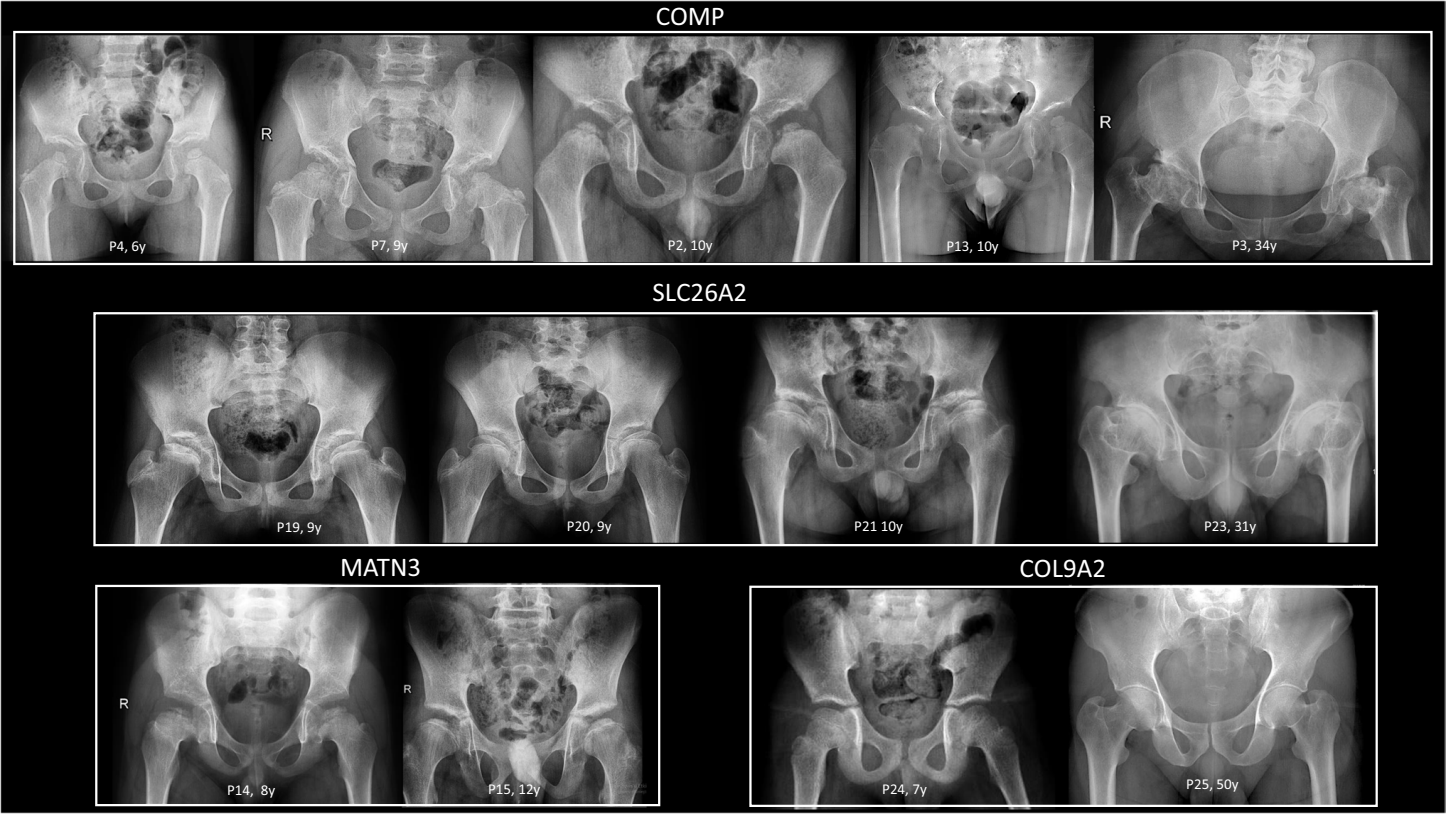
Pelvis radiographs of the patients. First row: Patients with *COMP* variants exhibit small, round epiphyses with irregular contours, except for P2, who has flattened and fragmented epiphyses (left). Coxa vara deformity with short and broad femoral necks is prominent in P7, P13, and P3. The ossified major trochanteric epiphyses are small and irregular in shape for P4 and P7. All patients present with dysplastic acetabula, and the adult patient (P3) has secondary osteoarthritis. Second row: Patients with *SLC26A2* variants show flattened epiphyses, and all have dysplastic acetabula. Coxa vara deformity, with short and broad femoral necks, is observed in all patients. Third row: Patients with *MATN3* variants have flattened epiphyses, and their femoral necks are short and broad, accompanied by mild acetabular dysplasia. The femoral epiphyses appear relatively normal in the patient with the *COL9A2* variant, while his father displays short femoral necks and coxa vara. P, patient; y, years

**Fig. 2:**
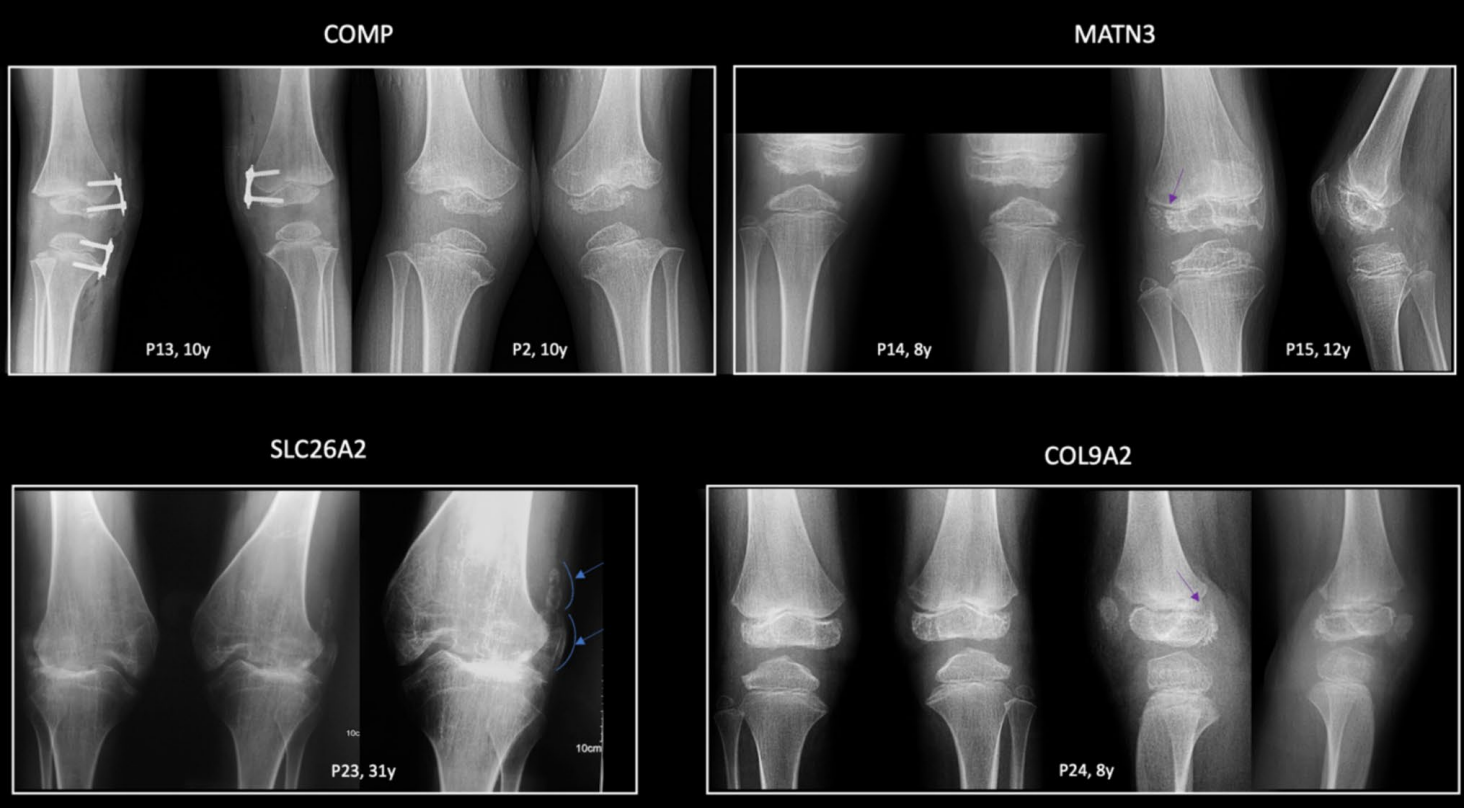
Knee radiographs of the patients. All knee epiphyses are small in patients with *COMP* and *MATN3* variants and irregularly shaped in P2, P14, and P15. P2 and P15 exhibit epiphyses with thin edges at the lateral borders. Additionally, flatness of the femoral condyles, with a shallow intercondylar notch, is observed in P14, P15, and P23. The typical finding of *SLC26A2*-related MED, bipartite patella, is seen in P23 and is indicated with blue lines and arrows. The glacier sign is present in P15 and P24, marked with a purple arrow. P, patient; y, years

**Fig. 3:**
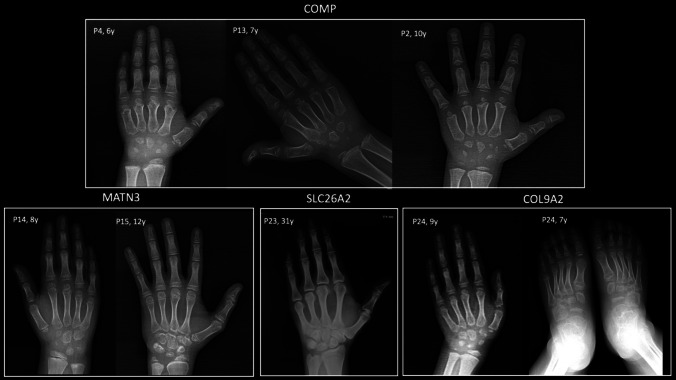
Hand radiographs of the patients and the foot radiograph of P24. All patients, except for the adult patient (P23), exhibit small and irregularly shaped carpal bones and epiphyses of the distal radius and ulna. Patients with *COMP* variants have short and broad metacarpal and phalangeal bones (brachydactyly), characterized by small, irregularly shaped epiphyses and metaphyseal irregularity. Patients with *MATN3* variants do not exhibit brachydactyly but also show mild contour irregularity of the carpal bones. Irregularly shaped tarsal bones are observed in P24, who has a *COL9A2* variant. P, patient; y, years

The clinical and genetic findings of the patients are summarized in Table 1. Pelvis, knee, and hand radiographs are shown in Figs. 1, 2, and 3, respectively. Of these 25 patients, 16 were male (64%) and nine were female (36%). The age at genetic diagnosis ranged from 4 to 50 years, with a median age of 10 years. Eight patients (*n* = 9/25, 36%) had short stature, 17 patients (*n* = 17/25, 68%) had joint pain, and seven patients (*n* = 7/25, 28%) required surgery.

**Table 1:** Clinical, radiographic, and molecular findings of the patients

**Family**	**Patient**	**Genetic findings**			**Radiographic findings**				**Clinical findings**					**Surgical intervention**
		**Gene**	**Variant**	**Genetic test**	**Spine**	**Pelvis**	**Knee**	**Hands**	**Gender**	**Age at admission (years)**	**Main complaint**	**Short stature**	**Pain**	
1	1	*COMP* NM_000095.3	c.1126 G>A p.(Asp376 Asn)	Sanger sequencing	Irregular endplates, anterior ossification defects	Epiphyseal irregularities	Epiphyseal irregularities	Normal	M	13	Walking difficulty	-	+	na
2	2				Normal	Epiphyseal irregularities	Genu valgum Epiphyseal irregularities	Normal	M	6	Short stature	+	+	+
	3				Scoliosis, increased lomber lordosis	Short femur neck	Genu varum	Short metacarpals	F	34	Short stature	+	-	-
3	4		c.1303 G>C p.(Asp435His) (*)	Sanger sequencing	Ovoid vertebral bodies	Small capital femoral epiphysis	Small and irregular epiphysis	Irregular-shaped carpal bones and epiphysis	F	6	Short stature, joint movement limitation, waddling gait, fatigue	+	-	-
4	5		c.1854 G>C p.(Glu618 Asp) (**)	Sanger sequencing	Mild vertebral irregularities	Epipyhseal irregularities	Epipyhseal irregularities	Normal	M	12	Joint pain	-	+	-
	6				Normal	Epipyhseal irregularities, short femur neck, coxa vara	Mild epiphyseal irregularities	Mildly short metacarpals Small epiphysis	M	7	Walking difficulty, joint pain	+	+	+
5	7		c.949 G>A p.(Asp317 Asn)	WES	Increased lomber lordosis, mild scoliosis	Short femur neck epiphyseal irregularities	Epipyhseal irregularities	Normal	F	11	Short stature, fatigue, hip dislocation	+	+	+
	8			Sanger sequencing	Severe scoliosis	Epipyhseal irregularities, acetabular dysplasia, short femur neck, small capital femoral epiphysis	Epipyhseal irregularities	Small and irregular-shaped carpal bones	F	7	Muscle weakness	-	-	+
	9			Sanger sequencing	na	na	na	na	M	39	Short stature, hip dislocation	+	-	na
6	10		c.1454 G>A p.(Arg485His)	WES	Normal	Epiphyseal irregularities	Epiphyseal irregularities small epiphysis	Small epiphysis, irregular-shaped carpal bones	F	9	Joint pain	-	+	-
	11			Sanger sequencing	Anterior ossification defect	Epiphyseal irregularities	Epiphyseal irregularities	Normal	F	11	Joint pain	-	+	-
	12			Sanger sequencing	na	na	na	na	M	41	na	Na	na	na
7	13		c.1467 C>A p.(Asn489Lys)	WES	-			Small and irregular-shaped carpal bones	M	8	Joint pain	+	+	+
8	14	*MATN3* NM_002381.5	c.518 C>A p.(Ala173 Asp)	Sanger sequencing	na	Epiphyseal irregularities	na	Irregular-shaped carpal bones	M	4	Walking difficulty	-	+	na
9	15		c.280 G>A p.(Val94Ile) (***)	WES	Increased lumbar lordosis	Small and irregulalar capital femoral epipyhisis	Small and irregular epiphysis, glacier crevice sign	Irregular-shaped carpal bones and epiphysis	M	12	Walking difficulty	-	+	-
	16			Sanger sequencing	Normal	Irregular femoral head, short femur neck	Normal	na	M	44	Joint pain	-	+	+
	17			Sanger sequencing	na	na	na	na	M	NA	na	Na	na	na
	18			Sanger sequencing	na	na	na	na	F	NA	na	Na	na	na
10	19	*SLC26A2* NM_000112.4	c.835 C>T p.(Arg279 Trp)	WES	Mild irregularities, scolisosis	Flat capital femoral epiphysis, coxa valga	Genu valgum	Normal	F	10	Joint pain	-	+	-
	20			Sanger sequencing	Normal	Flat capital femoral epiphysis	Genu valgum	Normal	F	10	Joint pain	-	+	-
11	21		c.1957 T>A p.(Cys653Ser)	Sanger sequencing	Mild platspondyly	Coxa vara, epiphyseal irregularities	Epiphyseal irregularities, bipartite patella	Normal	M	10	Joint pain, walking difficulty	+	+	na
	22				na	Acetabular dysplasia, flat capital femoral epiphysis	Double-layered patella	Normal	M	7	Joint movement limitation	-	+	na
	23				na	na	Double-layered patella	na	M	28	Joint movement limitation	Na	na	na
12	24	*COL9A2* NM_001852.4	c.186+6T>G	WES	na	na	Epiphyseal irregularities, glacier crevice sign	na	M	7	Joint pain, difficulty with climbing stairs	-	+	-
	25			Sanger sequencing	Decreased antero-posterior diameter	Short femoral necks	Genu varum; decreased joint space	Mild diaphyseal contruction of the phalanges and metacarpals	M	50	Joint pain, walking difficulty, genu varum	+	+	+

*na* not available, *WES* whole exome sequencing, *M* male, *F* female

^*^Novel variants

The orthopedic surgeries included corrective procedures for genu valgum in two patients (one patient had bilateral femur osteotomies, the other one had bilateral femur hemiepiphysiodesis); one patient had acetabular dysplasia and underwent Ganz periacetabular osteotomy; one patient with scoliosis had posterior instrumentation and fusion; two patients with proximal femoral deformity had corrective hip procedures, and the last one had total hip arthroplasty for end-stage hip osteoarthritis.

A variety of orthopaedic procedures were applied for the hip, spine, and lower extremity deformities in our patient population: Two patients had distal femoral correction osteotomies and plate fixation for genu valgum deformity. One patient with open growth plates had bilateral medial distal femur guided growth surgery utilizing tension band plates. The most common hip problems in this patient group are acetabular dysplasia and proximal femoral deformity. One of our patients had periacetabular pelvic osteotomy (Ganz) for the treatment of acetabular dysplasia, and two patients had proximal femoral osteotomies. One patient with end-stage hip osteoarthritis due to acetabular dysplasia had total hip arthroplasty surgery. One patient had posterior instrumentation and fusion for scoliosis deformity.

The surgical notes for the 50-year-old patient who underwent surgery during childhood could not be accessed; however, it was learned verbally that the surgery was performed due to curvature in the knees (genu varum deformity?).

**Fig. 4:**
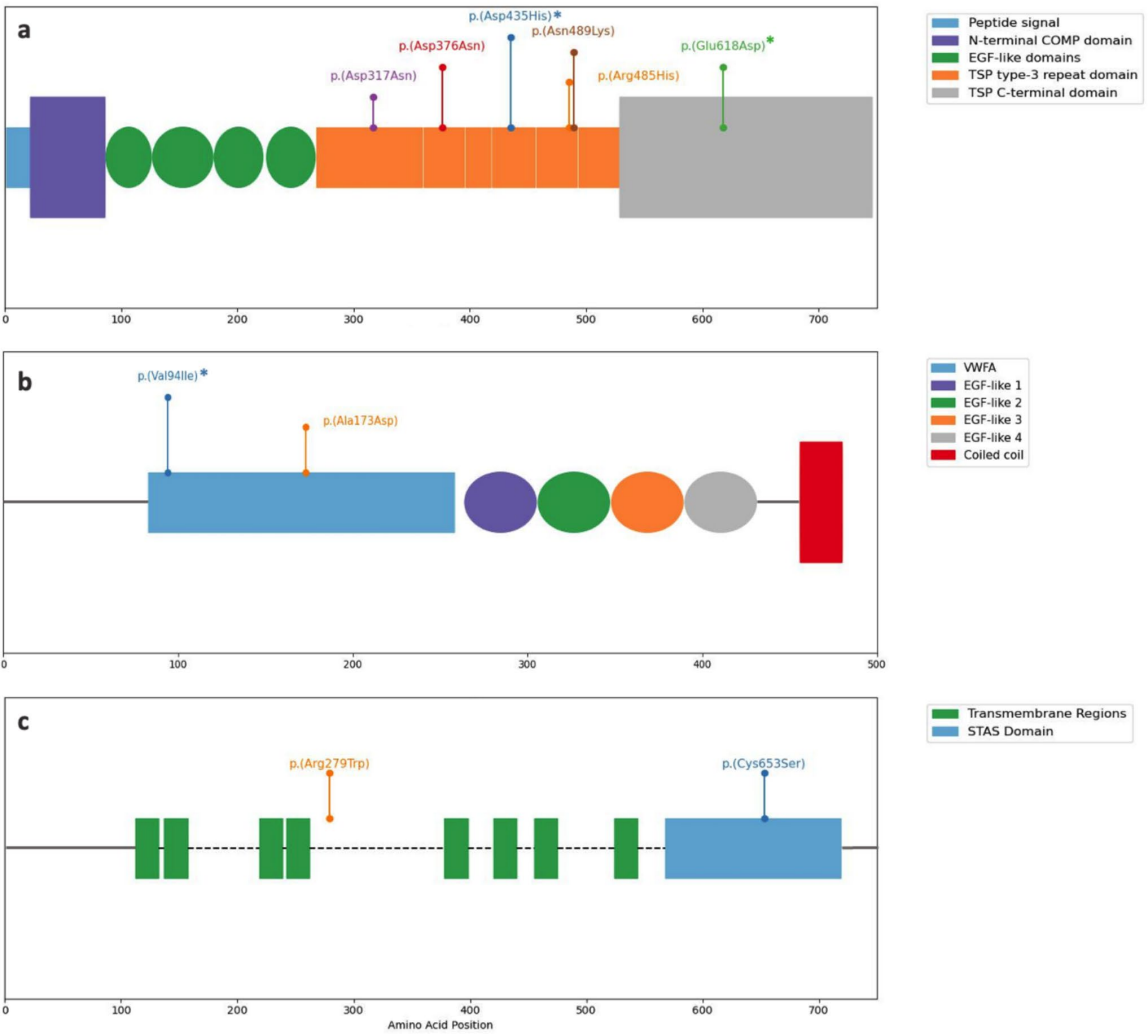
A schematic representation of the proteins and locations of the variants. COMP protein domains with 6 different missense variants identified in patients (**a**), MATN3 protein domains with two different missense variants identified in patients (**b**), and SLC26A2 protein domain and transmembrane regions with two different missense variants identified in patients (**c**). The domains are color-coded to indicate their functional regions, while the missense variants are marked with different colors to show their locations

**Fig. 5:**
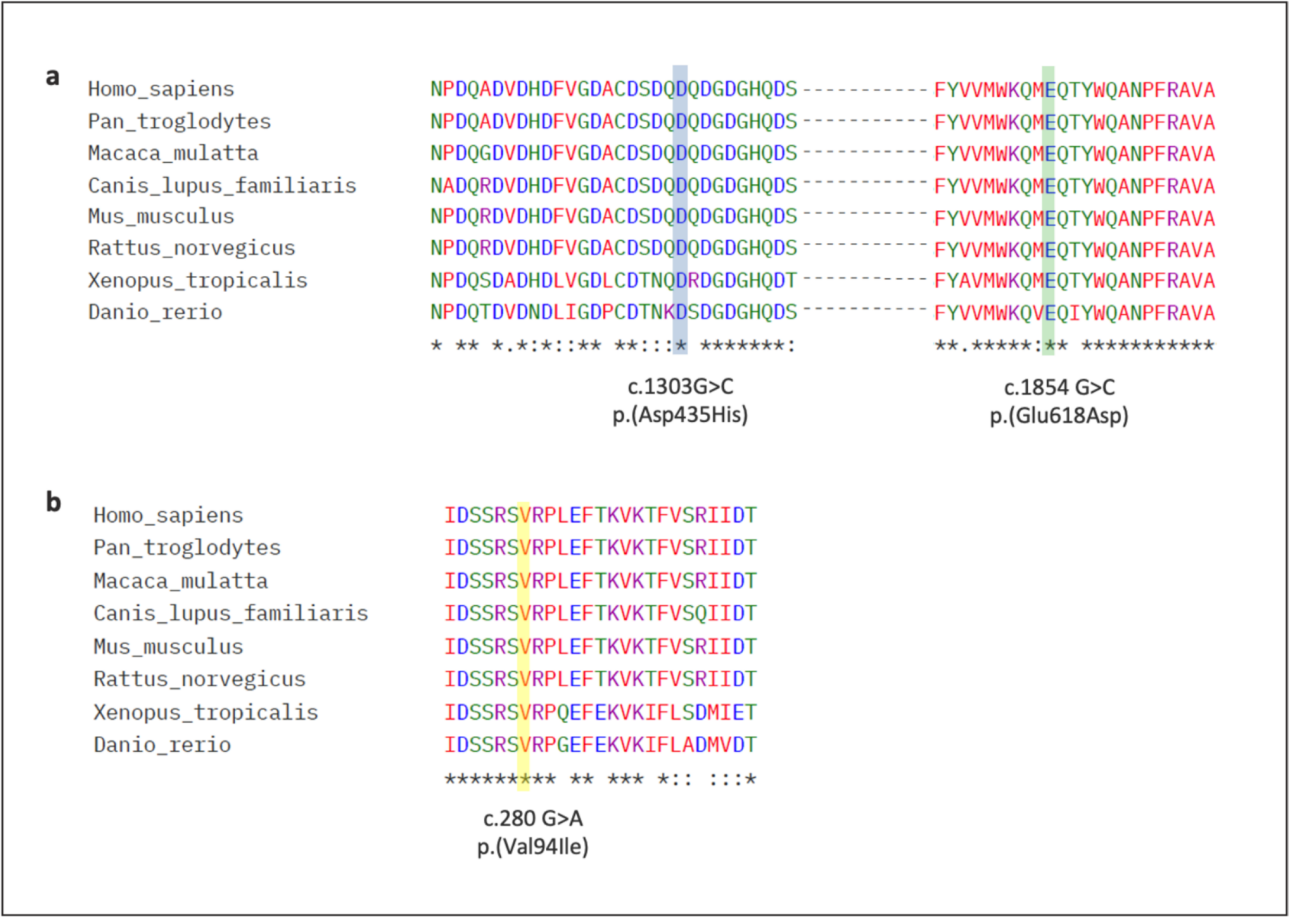
The amino acid multiple sequence alignment. COMP protein across different species. The alignment emphasizes the residues at positions 435 (Asp435) and 618 (Glu618), which are highlighted in blue and green, respectively (**a**). MATN3 protein across different species. The alignment emphasizes the residue at position 94 (Val94) which is highlighted in yellow (**b**)

In our cohort, we identified two novel variants in the COMP gene, c.1303 G > C; p.(Asp435His) and c.1854 G > C; p.(Glu618 Asp), and one novel variant in the MATN3 gene (c.280 G > A; p.(Val94Ile). According to the ACMG classification, the c.1303G > C variant was deemed “likely pathogenic” (PP3), with a CADD score of 34, PolyPhen2 predicting it as probably damaging, and Mutation Taster indicating it as disease-causing. On the other hand, the c.1854G > C variant was categorized as “uncertain significance” (PM1, PM2), with a CADD score of 23.1, PolyPhen2 predicting it as probably damaging, and Mutation Taster indicating it as disease-causing. Similarly, the c.280G > A variant in the MATN3 gene was also classified as “uncertain significance” (PM1, PM2), with a CADD score of 26.9, PolyPhen2 predicting it as probably damaging, and Mutation Taster indicating it as disease-causing. The three novel variants were located in the functional domains of the relevant proteins (Fig. 4) and the substituted amino acid residues were found to be evolutionarily conserved among different species (Fig. 5). We identified an intronic variant in *COL9A2* (c.186 + 6 T > G), affecting the splice donor site of intron 3–4. This variant has been previously reported to cause exon skipping, leading to the deletion of exon 3 in the transcript, and is associated with multiple epiphyseal dysplasia [20]. Based on ACMG guidelines, the variant was classified as a variant of uncertain significance (VUS). The variant was inherited from the affected father. Furthermore, it is absent from the gnomAD population database, fulfilling the PM2 criterion. Although classified as VUS, the presence of functional evidence supporting a splicing defect strengthens its potential relevance to the disease.

## Discussion

Multiple epiphyseal dysplasia (MED) is a relatively common skeletal dysplasia, with a prevalence estimated to be greater than 1 in 10,000 births [3]. Patients exhibit considerable clinical variability, and individuals with milder forms of MED may remain undiagnosed [3, 21]. Initial symptoms are often rheumatologic or neurologic, such as joint pain, difficulty walking, and signs of myopathy. Typically, patients appear normal at birth, but clinical manifestations emerge in early childhood, commonly presenting as joint pain. Although adult height is generally within the normal range, it may be mildly reduced. Joint pain tends to worsen progressively, and joint deformities may develop. This condition frequently leads to early-onset osteoarthritis, particularly affecting large weight-bearing joints [22].

MED is a genetically heterogeneous condition, with seven known genes implicated in its etiology, making it challenging to distinguish between the various subtypes based solely on radiologic features. However, certain distinguishing characteristics can aid in the diagnostic process. *COMP*-related MED (Type 1) is the most common form and accounts for up to half of all cases [3, 8]. In our study, the most frequent type was Type 1, with 13 of 25 patients (52%) from seven of 12 families (58.3%) carrying a *COMP* variant—findings that are consistent with the literature. Patients with Type 1 MED typically present with muscular hypotonia and mild myopathy, characterized by mildly elevated serum creatine kinase levels, genu varum, and joint laxity. These findings often lead to referrals to pediatric neurology due to delayed ambulation and suspicion of a neuromuscular disorder based on delayed motor development and increased serum creatine kinase levels [23–25].

Radiologic features associated with Type 1 MED include delayed carpal bone age, particularly more delayed than that of the phalangeal epiphyses, delayed ossification of the proximal femoral epiphyses, and small, rounded proximal femoral epiphyses (Figs. 1 and 2). Additional ossification centers may be present, with a distinct “glacier crevice” sign observed at the knees. The carpal bones often exhibit irregular contours (Fig. 3). Additional skeletal abnormalities, such as brachydactyly, small and rounded metacarpal epiphyses, ragged contours of tarsal bones, and mild spinal dysplasia—including endplate irregularities and mild platyspondyly—may also be seen [8, 26]. Furthermore, acetabular changes are common in *COMP*-related MED [22], and adults with this condition may exhibit coxa vara and shortened femoral necks (Fig. 1) [8].

Legg-Calve-Perthes disease, also known as Perthes disease, primarily affects the hip joint and is characterized by avascular necrosis of the femoral head, which can lead to deformity and potential arthritis in the affected hip. Patients with MED can sometimes be misdiagnosed as having Perthes disease, as both conditions may involve avascular necrosis of the femoral heads. However, it is important to note that MED is a distinct condition, characterized by abnormal development of the growth plates in multiple joints—unlike Perthes disease, which involves both hips in only 10 to 20% of cases [27]. While there may be similarities in the presentation of avascular necrosis in both Legg-Calve-Perthes disease and MED, the underlying pathophysiology and clinical features of these conditions are markedly different [22].

*MATN3*-related MED (Type 5) is the second most prevalent form of the condition, although it has been identified as the most common type in Western Asia populations [2, 26, 28]. In our study, *MATN3* variants were the second most common, along with *SLC26A2* variants. In *MATN3*-related MED, carpal bone ossification is delayed; however, the bones are not irregularly shaped, as in *COMP*-related MED (Fig. 3) [8]. Femoral and knee epiphyseal ossification is also delayed, and the proximal femoral epiphyses are less rounded than those in *COMP*-related MED [8]. A distinct “Harlequin hat” or “Dutch Wooden shoe” appearance of the distal femoral epiphyses, characterized by a triangular shape with thinning at the lateral sides, serves as a significant indicator for *MATN3*-related MED; however, this phenotype was not observed in our patients [2, 8, 26]. Additionally, vertical metaphyseal striations in the distal femora are commonly observed in this type [26]. The clinical presentation of *MATN3*-related MED is generally milder in comparison to *COMP*-related MED [22, 29]. Pathogenic variants in *COL9A1*, *COL9A2*, and *COL9A3* are responsible for Types 6, 2, and 3 MED, respectively. These subtypes are considered to be “rare” and represent the mildest forms of the condition [30–32]. In our cohort, we identified two patients from a single family with a *COL9A2* variant. Patients with *COL9*-related MED may exhibit myopathy findings similar to those seen in *COMP*-related MED [30]. Radiographically, knee epiphyses are more commonly affected in *COL9*-related MED, whereas the proximal femoral epiphyses are relatively spared [7, 8].

MED Type 4 is inherited in an autosomal recessive manner and is caused by biallelic variants in *SLC26A2*. Approximately half of affected individuals present with clubfoot and clinodactyly. Additionally, some patients may exhibit cystic ear swelling [4]. Notably, joint laxity is absent in *SLC26A2*-related MED, whereas joint contractures are a common feature. Genu valgum (knock-knee deformity) is a prevalent manifestation of *SLC26A2*-related MED and is often accompanied by mild brachydactyly. Radiographic evaluation reveals flat epiphyses, a distinguishing feature compared to the rounded epiphyses observed in *COMP*-related MED. Furthermore, carpal bone age is typically advanced in *SLC26A2*-related MED in contrast to findings in *COMP*-MED [8]. A characteristic radiographic finding associated with *SLC26A2*-related MED is the presence of a double-layered patella, observed in approximately 60% of affected individuals [33]. While this finding is specific to *SLC26A2*-related MED, it is not highly sensitive and may also be seen in other MED types [4, 26, 34]. In some cases, variations such as a bipartite patella may be observed, as was demonstrated in our cohort.

Recent research has identified a seventh subtype of MED associated with biallelic pathogenic variants in the CANT1 gene (OMIM #617719). To the best of our knowledge, only two studies have been reported to date, one of which originated from our center [6, 35]. Patients with this condition often present with distinctive features, such as a monkey-wrench appearance of the proximal femora, advanced carpal and tarsal bone age, joint dislocations, and osteopenia. While these manifestations overlap with those observed in the allelic disorder Desbuquois dysplasia (DBQD) and the Kim variant, the overall severity of symptoms in this newly identified subtype tends to be milder than in DBQD [6, 35].

The etiology of MED involves seven identified genes. However, a significant proportion of patients, estimated at 15–20%, remain without a genetic diagnosis, suggesting potential associations with as-yet-unidentified genetic factors [6]. In our cohort, a genetic etiology was identified in 12 out of 14 families, aligning with existing literature and accounting for 85.7% of cases. This diagnostic rate may be influenced by several factors, including intronic variants, intragenic copy number variations, and complex structural rearrangements within known genes. Additionally, undiagnosed cases may be attributable to genes not yet been implicated in the pathogenesis of MED.

We observed significant variability in clinical and radiologic findings among family members, even within the same family in our cohort. The variability in clinical presentation among affected individuals is a well-documented phenomenon in genetic disorders, including MED. This variability can arise from several factors, such as differences in genetic backgrounds, environmental influences, and the presence of modifier genes. Additionally, our findings align with existing literature that highlights the complexity of genetic disorders. The diverse clinical manifestations observed in our cohort underscore the need for a comprehensive understanding of how genetic and environmental factors interact to influence the phenotypic expression of MED.

The limitations of our study include the incomplete segregation analysis for patients whose parental DNA could not be obtained, as well as the lack of experimental functional validation for the three novel variants identified, which were supported by in silico analyses indicating pathogenicity. In the absence of experimental data, it is important to explicitly recognize this gap and suggest that future research should focus on functional studies to validate these novel variants. Additionally, further research and advances in genetic analysis techniques are essential for uncovering these elusive genetic contributors and expanding our understanding of the etiology of MED.

## Conclusion

Multiple epiphyseal dysplasias encompass a diverse group of genetically heterogeneous disorders, resulting in variable clinical and radiological features. It is important for healthcare providers to consider multiple epiphyseal dysplasias in the differential diagnosis of patients presenting with joint pain, walking difficulties, or symptoms resembling Perthes disease or myopathy. A multidisciplinary approach, involving professionals from various specialties, is essential for the effective management of MEDs. Additionally, ongoing orthopedic follow-up is crucial for monitoring disease progression and addressing the specific needs of these patients. As new genes associated with MEDs are identified and more cases are reported, a more accurate and comprehensive approach to diagnosis and management will continue to evolve.

## Data Availability

No datasets were generated or analysed during the current study.

## Abbreviations

CANT1: Calcium-activated nucleotidase 1
COMP: Cartilage oligomeric matrix protein
DBQD: Desbuquois dysplasia
MATN3: Matrillin 3
SLC26A2: Solute carrier family 26 member 2
COL9A1/COL9A2/COL9A3: Collagen type 9 A1/A2/A3
MED: Multiple epiphyseal dysplasia
